# Synergistic effects of colchicine combined with atorvastatin in rats with hyperlipidemia

**DOI:** 10.1186/1476-511X-13-67

**Published:** 2014-04-17

**Authors:** Congwu Huang, Chuan Cen, ChengXu Wang, Haiyong Zhan, Xin Ding

**Affiliations:** 1 The Department of Internal Medicine, the Second Affiliated Hospital of Shantou University, Shantou, China; 2 The Department of Internal Medicine, Zhongshan People’s Hospital, Zhongshan, China; 3 The Emergency Department, the Second Affiliated Hospital of Dalian Medical University, Dalian, China

**Keywords:** Hyperlipidemia, Inflammation, Endothelial dysfunction

## Abstract

**Background:**

Inflammation and endothelial dysfunction is implicated in the atherosclerosis initiation and progression in the setting of hyperlipidemia. Colchicine is a potent anti-inflammatory agent and whether colchicine combined with atorvastatin has synergistic effects on inflammation amelioration and endothelial function improvement is unknown.

**Methods:**

Hyperlipidemic rat model was produced by high-fat and high-cholesterol diet for 6 weeks. Rats with normal diet were served as shame group. In hyperlipidemic group, normal saline, atorvastatin (10 mg/kg body weight/day), colchicines (0.5 mg/kg body weight/day), or atorvastatin combined with colchicines (same dosages) were prescribed for 2 weeks. Serum levels of lipid profile, C-reactive protein (CRP), liver enzyme, lipoprotein associated phospholipase A2 (Lp-PLA2) and nitric oxide (NO) production were serially assessed.

**Results:**

Before the beginning of the study, all laboratory variables were comparable among each group. After 6 weeks of hyperlipidemic model production, serum levels of cholesterols, CRP and Lp-PLA2 were significantly increased when compared to sham group, whereas NO production was reduced. With 2 weeks of colchicine therapy, serum levels of CRP and Lp-PLA2 were decreased and NO production was enhanced in the colchicine group in a lipid-lowering independent manner. Added colchicine into atorvastatin therapy further improved NO production and decreased CRP and Lp-PLA2 levels, indicating a potential synergism of colchicine and atorvastatin.

**Conclusion:**

Colchicine combined with atorvastatin may have stronger protective effects on improving endothelial function and ameliorating inflammation in rats with hyperlipidemia.

## Introduction

Hyperlipidemia is a major cause of multiple diseases such as atherosclerotic cardiovascular diseases (CVD) [1,2]. The mechanisms of hyperlipidemia implicated in the initiation and progression of CVD predominantly involve sustained endothelial dysfunction and vascular inflammation [3-5]. Previously, many animal studies and clinical trials also have consistently demonstrated that with statins therapy, a potent agent in regulating lipid metabolism, not only lipid profile disorder has been corrected but also systemic inflammation is ameliorated as indicated by the decrease of inflammatory cytokines such as C-reactive protein (CRP) [6-9].

Lipoprotein associated phospholipase A2 (Lp-PLA2) is a key enzyme responsible for degrading platelet-activating factor (PAF) and oxidated-LDL (ox-LDL). Initially, some basic studies showed that Lp-PLA2 was beneficial for deterring atherosclerosis progression by means of degrading PAF, a potent pro-inflammatory cytokine [10-12]. Nevertheless, thereafter, a large number of clinical and experimental studies have consistently revealed that increased Lp-PLA2 level was associated with increased risk of cardiovascular events [13-15], which was considered to be associated with the increased production of lyso-phosphotidylcholine (Lyso-PC) and oxidized non-esterified fatty acids (oxNEFAs), two potent pro-inflammatory and pro-atherosclerotic intermediates derived from ox-LDL degradation by Lp-PLA2 [16,17]. Notably, some studies showed that statins might have effects on reducing Lp-PLA2 level [18-20], nonetheless, other studies showed no favorable effects of statins on Lp-PLA2 reduction [21,22]. Therefore, whether statins can reduce Lp-PLA2 is still inconclusive.

Colchicine is an old medicine and has been used for gout and other inflammatory diseases due to its potent effect on improving inflammatory reactions. Recently, a study conducted by Nidorf and colleagues showed that colchicine combined with statins was beneficial for cardiovascular events’ prevention [23]. The underlying mechanisms are far from clear, however. Previously, one study revealed that colchicine could inhibit adhesion of neutrophilic granulocytes to epidermal sections induced by PAF [24]. Since most of circulating Lp-PLA2 is produced by macrophages within vascular wall [17], therefore, we hypothesized that colchicine might reduce Lp-PLA2 production through inhibiting leukocytes adhesion and infiltration.

Taken together, in light of the crucial roles Lp-PLA2 plays on the initiation and progression of vascular inflammation and atherosclerosis in subjects with hyperlipidemia and the potent effect of colchicine on regulating inflammation, we hypothesized that colchicine might be effective in ameliorating vascular inflammation and improving endothelial function by means of declining Lp-PLA2 level, and if corroborated, we believed that in the future adds colchicines into statins therapy may have additional benefits on CVD prevention and therapy.

## Methods

### Animal model and study protocol

Male Sprague–Dawley (SD) rats weighing 200-220 g were obtained from Experimental Animal Center of Shantou University, Shantou, China. The study was approved by Ethic Committee of Shantou University. Totally 50 rats were used in current study and after 1 week’s accommodation were evenly and randomly divided into 5 groups. Ten rats given normal diet were served as sham group, and the other 40 rats were given a high-fat and high-cholesterol diet as described by previous study with mild modification (cholesterol 4%, cholic acid 0.4%, propylthiouracilum 0.3% and lard 10%) for 6 weeks for hyperlipidemic model production [25]. Subsequently, the 40 hyperlipidemic rats were randomly and evenly assigned into 4 groups as follow: control group was orally given normal saline, statins group orally given atorvastatin (10 mg/kg body weight/day, reconstituted in normal saline), colchicine group intraperitoneally injected colchicine (0.5 mg/kg body weight/day, dissolved in 0.05% dimethyl sulfoxide, DMSO), and combined group given atorvastatin and colchicine as described above. Total intervention duration was 2 weeks.

### Laboratory examination

Fasting blood was taken for laboratory examination before the beginning of the study, after 6 weeks of model production, and after 2 weeks of intervention. The variables for examination involved serum levels of triglyceride (TG), total cholesterol (TC), low density lipoprotein cholesterol (LDL-C), high density lipoprotein cholesterol (HDL-C), alanine aminotransferase (ALT), aspartate aminotransferase (AST) and CRP, which were assessed by Automatic Biochemistry Analyzer (Hitachi 7150, Tokyo, Japan). Serum level of nitric oxide (NO) was evaluated by nitrite reductase method using Total Nitric Oxide Kit (Beyotime, Haimen, China, S0023), and serum level of Lp-PLA2 was assessed by sandwich enzyme-linking immune-sorbent assay (ELISA) kit (Yanjin Biochemistry Company, Shanghai, China). Three independent experiments were performed in duplicate.

### Statistical analyses

All continuous variables were expressed as mean ± SD, and analyses were performed with SPSS software, version 18.0 (SPSS Science, Chicago, IL, USA). Statistical significance among groups was evaluated with One Way ANOVA (post hoc LSD-t), and a value of *P* < 0.05 was considered statistically significant.

## Results

### Changes of lipid profile and other variables

As presented in Table 1, the baseline laboratory variables among different groups were comparable at the very beginning, and after 6 weeks of high-fat and high-cholesterol diet administration, the serum levels of TG, TC and LDL-C in hyperlipidemic model groups were significantly increased when compared to the sham group. Additionally, serum level of CRP was also profoundly increased in hyperlipidemic model groups, indicating that hyperlipidemia was significantly associated with systemic inflammation. After two weeks’ intervention, serum levels of TC and LDL-C in the atorvastatin and combined groups were significantly reduced and no changes were found in the control and colchicine groups. When compared to the atorvastatin group, CRP reduction was more prominent in the colchicine group (5.35 ± 0.93 mg/L versus 4.03 ± 0.65 mg/L, P < 0.05), indicating that colchicine might have more robust effect on ameliorating inflammation than atorvastatin, which was independent of lipid-lowering. Importantly, this anti-inflammatory effect of colchicine was further enhanced when combined with atorvastatin as evidenced by the magnitude of CRP reduction in the combined group was more prominent than the other groups. Notably, no liver toxicity was found in each group, indicating that current used dosage of atorvastatin and/or colchicine was safe for 2 weeks’ therapy in rats.

**Table 1:** Changes of lipid profile and other variables (n = 10, each group)

**Variables**	**Sham**	**Control**	**Atorvastatin**	**Colchicine**	**Combined**
**Before the beginning of the study**					
**TG (mmol/L)**	0.93 ± 0.11	0.98 ± 0.14	0.93 ± 0.12	0.95 ± 0.10	0.96 ± 0.15
**TC (mmol/L)**	3.04 ± 0.35	3.11 ± 0.32	3.08 ± 0.27	3.06 ± 0.30	3.09 ± 0.33
**LDL-C (mmol/L)**	1.99 ± 0.24	1.98 ± 0.21	2.01 ± 0.29	1.97 ± 0.19	2.01 ± 0.24
**HDL-C (mmol/L)**	1.03 ± 0.08	1.03 ± 0.07	1.05 ± 0.07	1.04 ± 0.06	1.04 ± 0.05
**CRP (mg/L)**	1.24 ± 0.16	1.27 ± 0.13	1.32 ± 0.16	1.25 ± 0.19	1.24 ± 0.13
**ALT (U/L)**	24.5 ± 3.8	25.8 ± 3.0	25.2 ± 1.9	24.2 ± 1.7	26.0 ± 2.8
**AST (U/L)**	28.3 ± 2.3	27.6 ± 2.7	28.2 ± 2.4	29.1 ± 3.2	28.8 ± 2.5
**6 weeks of model production**					
**TG (mmol/L)**	0.98 ± 0.13*	2.15 ± 0.33	2.22 ± 0.37	2.19 ± 0.28	2.20 ± 0.27
**TC (mmol/L)**	3.08 ± 0.32*	5.87 ± 0.66	5.93 ± 0.61	5.91 ± 0.57	5.89 ± 0.60
**LDL-C (mmol/L)**	1.97 ± 0.21*	3.76 ± 0.55	3.79 ± 0.53	3.80 ± 0.51	3.78 ± 0.52
**HDL-C (mmol/L)**	1.05 ± 0.06	1.07 ± 0.04	1.08 ± 0.02	1.07 ± 0.04	1.06 ± 0.03
**CRP (mg/L)**	1.30 ± 0.13*	7.25 ± 1.08	7.20 ± 1.03	7.18 ± 1.08	7.22 ± 1.05
**ALT (U/L)**	27.8 ± 3.2	29.2 ± 3.6	28.5 ± 2.4	26.2 ± 1.4	26.8 ± 2.0
**AST (U/L)**	28.2 ± 3.5	28.2 ± 2.5	28.7 ± 2.0	28.3 ± 2.7	27.4 ± 1.9
**2 weeks of therapy**					
**TG (mmol/L)**	0.98 ± 0.13*	2.13 ± 0.25	1.98 ± 0.16	2.11 ± 0.22	1.97 ± 0.16
**TC (mmol/L)**	3.05 ± 0.24*	5.65 ± 0.48^#^	4.85 ± 0.41	5.85 ± 0.55^&^	4.64 ± 0.43
**LDL-C (mmol/L)**	1.93 ± 0.20*	3.70 ± 0.51^#^	2.92 ± 0.22	3.71 ± 0.38^&^	2.88 ± 0.25
**HDL-C (mmol/L)**	1.05 ± 0.03	1.06 ± 0.05	1.11 ± 0.03	1.08 ± 0.03	1.12 ± 0.03
**CRP (mg/L)**	1.32 ± 0.12*	7.19 ± 1.02^#^	5.35 ± 0.93	4.03 ± 0.65^&^	2.87 ± 0.45^※^
**ALT (U/L)**	26.3 ± 4.1	27.8 ± 1.4	28.2 ± 2.6	27.4 ± 1.8	26.3 ± 2.5
**AST (U/L)**	27.4 ± 3.0	27.6 ± 2.1	28.2 ± 2.4	28.0 ± 2.1	27.3 ± 1.4

Denote: *P < 0.05 versus other groups; ^#^P < 0.05 versus Atorvastatin and Combined groups; ^&^P < 0.05 versus Atorvastatin and Combined groups; ^&^P < 0.05 versus Control, Atorvastatin and Colchicine groups.

### Changes of NO production and serum level of Lp-PLA2

As presented in Table 2, after 6 weeks of high-fat and high-cholesterol diet administration, NO production in the hyperlipidemic model groups were significantly abolished when compared to the sham group, whereas serum levels of Lp-PLA2 were significantly elevated, indicating that hyperlipidemia might not only contribute to enhanced inflammation but also impaired endothelial function. After 2 weeks of treatment, NO production in the atorvastatin, colchicine and combined groups were increased when compared to the control group, and additionally the serum levels of Lp-PLA2 were concomitantly decreased. When compared to the atorvastatin group, the magnitude of Lp-PLA2 reduction was a little bit larger in the colchicine group, although there was no significant difference (143.2 ± 8.6 ng/mL versus 148.3 ± 9.9 ng/mL, P = 0.063), and NO production between atorvastatin and colchicine groups was also comparable (6.2 ± 0.7 μmol/L versus 6.1 ± 0.4 μmol/L, P = 0.106). Notably and interestingly, not only NO production was more profoundly increased, but Lp-PLA2 reduction was also more prominent in the combined group in comparison of the other hyperlipidemic groups, indicating that the combination of atorvastatin and colchicine had synergistic effects in rats with hyperlipidemia.

**Table 2:** Changes of NO production and serum level of Lp-PLA2

**Variables**	**Sham**	**Control**	**Atorvastatin**	**Colchicine**	**Combined**
**Before the beginning of the study**					
**NO (μmol/L)**	8.6 ± 0.7	8.9 ± 0.7	9.2 ± 0.5	9.0 ± 0.6	8.8 ± 0.6
**Lp-PLA2 (ng/mL)**	73.6 ± 6.7	78.7 ± 7.3	74.5 ± 6.6	76.2 ± 6.9	76.0 ± 5.7
**6 weeks of model production**					
**NO (μmol/L)**	8.2 ± 0.4*	4.3 ± 0.5	4.1 ± 0.4	4.4 ± 0.6	4.5 ± 0.6
**Lp-PLA2 (ng/mL)**	77.2 ± 8.1*	164.2 ± 13.5	168.7 ± 11.5	170.3 ± 15.2	166.8 ± 14.3
**2 weeks of therapy**					
**NO (μmol/L)**	8.9 ± 0.5	4.4 ± 0.6^#^	6.2 ± 0.7	6.1 ± 0.4	7.3 ± 0.3^&^
**Lp-PLA2 (ng/mL)**	80.1 ± 9.6	160.6 ± 10.2^#^	148.3 ± 9.9	143.2 ± 8.6	130.2 ± 5.8^&^

Denote: *P < 0.05 versus other groups; ^#^P < 0.05 versus Atorvastatin, Colchicine and Combined groups; ^&^P < 0.05 versus Control, Atorvastatin and Colchicine groups.

## Discussion

Our current study shows that in rats with hyperlipidemia, an early stage of atherosclerosis, colchicine therapy alone is potential in simultaneously ameliorating inflammation and improving endothelial function, which is independent of lipid-lowering. The effects of CRP and Lp-PLA2 reduction and NO production are further enhanced when atorvastatin combined with colchicine therapy, indicating that these two drugs may have synergistic benefits on deterring atherogenesis and atherosclerotic progression.

Hyperlipidemia, which is characterized by increased LDL-C and TC levels and/or decreased HDL-C level, is a major risk factor for CVD. The underpinning mechanisms by which hyperlipidemia contributing to atherogenesis and atherosclerotic progression involve impairing endothelial function and eliciting endothelial activation, increasing foam cells formation, and enhancing vascular inflammation [4,26,27]. Therefore, treating hyperlipidemia with medications such as statins is favorable for ameliorating vascular inflammation, improving endothelial function and deterring atherosclerosis. As shown in our current study, in rats with hyperlipidemia producing by high-fat and high-cholesterol diet, 2 weeks of atorvastatin (10 mg/kg/day) therapy significantly improved dyslipidemia and reduced CRP level, which was consistent with previous finding [9]. Expectedly, the traditional potent anti-inflammatory medicine colchicine also had a robust effect on declining serum CRP level, which was independent of lipid-lowering. As is well known that, increased CRP level is a significant risk factor for adverse cardiovascular outcomes as strongly supported by the JUPITER trial in which, when compared to placebo therapy, rosuvastatin significantly reduces adverse cardiovascular events in participants with normal lipid profile but with increased CRP level [28]. Our current study showed that the efficacy of statins on CRP reduction was further enhanced by colchicine addition suggesting that these two medications may have synergistic effects on improving systemic inflammation and may also have additional benefits for cardiovascular events prevention.

As is well documented that endothelial dysfunction, in terms of reduced NO production, plays critical roles on atherosclerosis initiation and progression [29], and additionally, as Lp-PLA2 elevation has been recognized as a new target for therapy in patients with CVD [30], we further investigated the effects of colchicine on endothelial function and Lp-PLA2 level. Previously, some studies have been conducted to evaluate the effects of statins on serum Lp-PLA2 level, and the results indicated that statins had modest effect on Lp-PLA2 reduction, and currently two large randomized controlled phase-III clinical trials (STABILITY and SOLID-TIMI 52) are ongoing to evaluate the efficacies of darapladib (a specific inhibitor for Lp-PLA2) on cardiovascular outcomes, which we believe may shift the paradigm of CVD therapy in the future due to the highly sensitive and specific characteristic of Lp-PLA2 for vascular inflammation. The results from our current study indicated that colchicine has a marginally better effect on Lp-PLA2 reduction than atorvastatin (143.2 ± 8.6 ng/mL versus 148.3 ± 9.9 ng/mL, P = 0.063). In light of the mechanisms of actions of colchicine [31], we speculated that the efficacy of colchicine on Lp-PLA2 reduction might be at least partially associated with its effects on inhibiting inflammatory cells migration and infiltration. Since circulating Lp-PLA2 is largely produced by macrophages within vascular wall, therefore, inhibiting leukocytes adhesion and activation by colchicine was favorable for reducing Lp-PLA2 production. Additionally, increased NO production, which we considered derived from vascular inflammation amelioration, by colchicine therapy might reciprocally contribute to Lp-PLA2 production. Since NO could diminish oxidative stress and reduce ox-LDL production, which in turn leads to decrease foam cells formation and Lp-PLA2 excretion by macrophages and foam cells [29,32]. Taken together, we believed that colchicine reducing Lp-PLA2 production was dependent on its effects on ameliorating inflammation and improving endothelial function. Importantly, NO production and Lp-PLA2 reduction were more prominent in colchicine combined with atorvastatin therapy, indicating that adding colchicine to statins therapy might further enhance the protective effects of statins therapy. These mechanisms might at least partially explain the protective effect of statins combined with colchicine therapy on reducing cardiovascular events in patients with stable chronic coronary artery disease [23,33]. However, since the animal model of our current study was a simple scenario in terms of only having hyperlipidemia, whether colchicine really has an amazing and synergistic effect on more complicated conditions such as metabolism syndrome ensuing acute myocardial infarction in which endothelial function maybe already irreversible and inflammatory cascade within atherosclerotic plaque maybe already uncontrollable needs to be further investigated.

Finally, with regard to the potential side effects of colchicine combined with statins therapy, serum level of liver enzymes such ALT and AST were evaluated before and after therapy, and without any significant increase of liver enzymes was found. However, because our current study has not detected the changes of creatinine kinase levels, we cannot exclude the potential myopathy incidence induced by colchicine combined with statins therapy. Therefore, in the future to investigate whether colchicine combined with statins would increase the risk of myopathy is of particular importance.

## Conclusion

Results from our current study show that in rats with hyperlipidemia, colchicine therapy is beneficial for reducing CRP level, increasing NO production and decreasing Lp-PLA2 level, which is independent of lipid-lowering. Colchicine combined with atorvastatin therapy has synergistic effects on improving endothelial function and ameliorating inflammation which we believe may be helpful and beneficial for future studies in exploring optimal therapeutic strategies for atherosclerosis and CVD preventions in the setting of hyperlipidemia.

## Competing interests

The authors declare that they have no competing interests.

## Authors’ contribution

CC, CW and XD performed this study, HZ performed statistic analyses, and CH designed this study and wrote this article. All authors read and approved the final manuscript.

## Authors’ information

Congwu Huang and Chuan Cen co-first authors.
